# SZR-104, a Novel Kynurenic Acid Analogue with High Permeability through the Blood–Brain Barrier

**DOI:** 10.3390/pharmaceutics13010061

**Published:** 2021-01-05

**Authors:** Kinga Molnár, Bálint Lőrinczi, Csilla Fazakas, István Szatmári, Ferenc Fülöp, Noémi Kmetykó, Róbert Berkecz, István Ilisz, István A. Krizbai, Imola Wilhelm, László Vécsei

**Affiliations:** 1 Institute of Biophysics, Biological Research Centre, 6726 Szeged, Hungary; molnar.kinga@brc.hu (K.M.); fazakas.csilla@brc.hu (C.F.); krizbai.istvan@brc.hu (I.A.K.); 2 Theoretical Medicine Doctoral School, University of Szeged, 6720 Szeged, Hungary; 3 Institute of Pharmaceutical Chemistry and Research Group for Stereochemistry, Hungarian Academy of Sciences, University of Szeged, 6720 Szeged, Hungary; lorinczi.balint@pharm.u-szeged.hu (B.L.); szatmari.istvan@pharm.u-szeged.hu (I.S.); fulop@pharm.u-szeged.hu (F.F.); 4 Institute of Pharmaceutical Chemistry, Interdisciplinary Excellence Center, University of Szeged, 6720 Szeged, Hungary; 5 Institute of Pharmaceutical Analysis, Interdisciplinary Excellence Center, University of Szeged, 6720 Szeged, Hungary; kmetyek13@gmail.com (N.K.); berkecz.robert@pharm.u-szeged.hu (R.B.); ilisz@pharm.u-szeged.hu (I.I.); 6 Institute of Life Sciences, Vasile Goldiş Western University of Arad, 310025 Arad, Romania; 7 Department of Neurology, Interdisciplinary Excellence Center, University of Szeged, 6725 Szeged, Hungary; 8 MTA-SZTE Neuroscience Research Group, Hungarian Academy of Sciences, University of Szeged, 6725 Szeged, Hungary

**Keywords:** aminoalkylated amide derivatives, blood–brain barrier (BBB), in vitro model, kynurenic acid (KYNA), permeability, SZR-104

## Abstract

By being an antagonist of glutamate and other receptors, kynurenic acid serves as an endogenous neuroprotectant in several pathologies of the brain. Unfortunately, systemic administration of kynurenic acid is hindered by its low permeability through the blood–brain barrier. One possibility to overcome this problem is to use analogues with similar biological activity as kynurenic acid, but with an increased permeability through the blood–brain barrier. We synthesized six novel aminoalkylated amide derivatives of kynurenic acid, among which SZR-104 (*N*-(2-(dimethylamino)ethyl)-3-(morpholinomethyl)-4-hydroxyquinoline-2-carboxamide) proved to have the highest permeability through an in vitro blood–brain barrier model. In addition, permeability of SZR-104 was significantly higher than that of kynurenic acid, xanthurenic acid and 39B, a quinolone derivative/xanthurenic acid analogue. Since peripherally administered SZR-104 is able to inhibit epileptiform activity in the brain, we conclude that SZR-104 is a promising kynurenic acid analogue with good penetrability into the central nervous system.

## 1. Introduction

**Figure 1:**
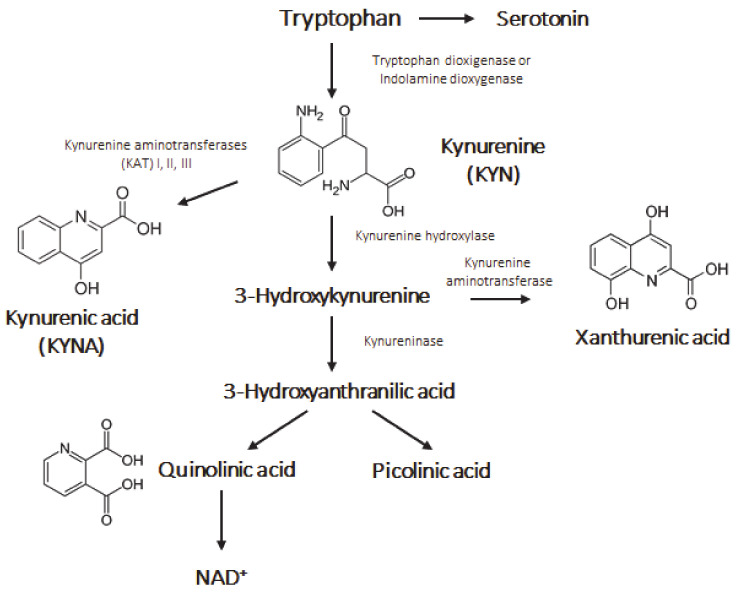
The kynurenine pathway**.**

The kynurenine (KYN) pathway plays an important role in the production of nicotinamide adenine dinucleotide (NAD+), nicotinamide adenine dinucleotide phosphate (NADP+) and other notable compounds from the cleavage of tryptophan (Trp) [1]. As a result of this diverse, multiple branched cleavage pathway, 95% of Trp is converted into kynurenic acid (KYNA) (Figure 1). KYNA is a well-known endogenous neuromodulator that controls the level of several neurotransmitters, such as glutamate, acetylcholine, dopamine and γ-aminobutyric acid (GABA). By being a non-competitive antagonist at the glycine site of glutamatergic N-methyl-D-aspartate (NMDA) receptor, KYNA is able to decrease excitotoxicity and serves as an endogenous neuroprotectant.

Besides, KYNA is a weak antagonist on kainate and α-amino-3-hydroxy-5-methyl-4-isoxazolepropionic acid (AMPA) glutamatergic and a few other receptors. Moreover, KYNA also acts as a free radical scavenger. All these effects complement the potent neuroprotective profile of KYNA [2]. An increasing amount of data regarding KYN metabolites suggests their indisputable role in neurophysiological diseases, such as ischaemia, headache, schizophrenia, epilepsy, Alzheimer’s disease (AD), Huntington’s disease (HD), Parkinson’s disease (PD), amyotrophic lateral sclerosis (ALS) and multiple sclerosis (MS). It has been shown that levels of KYNA drop in the plasma and in red blood cells of AD patients [3] as also in cerebrospinal fluid (CSF) of relapsing-onset MS patients during remission [4]. In the animal model of depression, decreased levels of KYNA were observed in the prefrontal cortex [5], while in HD patients, low levels of KYNA and reduced activity of kynurenine aminotransferase (KAT) were detected in the striatum and cortex [6,7,8]. These results arise the question whether exogenous application of KYNA could be beneficial in the treatment of neurological diseases. Unfortunately, KYNA has a very limited ability to pass the blood–brain barrier (BBB). By forming an active interface between the blood and the central nervous system (CNS), the BBB limits the permeability of several substances into the brain tissue. Formation of the BBB is one of the main functions of the neurovascular unit (NVU), endothelial cells of the brain being the most restrictive in the organism. Continuous tight junctions, efflux transporters, metabolic enzymes and other mechanisms contribute to the low permeability of the BBB, which not only restricts passage of harmful molecules, but also of several therapeutic agents [9]. Thus, pharmacological approaches are urgently needed to overcome the issue of low penetration of KYNA into the brain and to achieve its therapeutic potential [2,10]. There are three main possibilities that can be considered: first, administration of the BBB-penetrant KYNA precursor, KYN and its halogenated derivates. Fukushima and colleagues presented a comparative study of intraperitoneally administered natural isomer L-KYN and the unnatural D-KYN in plasma. Majority of L-KYN was rapidly metabolized by kynurenine-3-hydroxylase and kynureninase into 3-hydroxykynurenine and anthranilic acid and not into KYNA by KAT I and KAT II in the tissue, while D-KYN was metabolized into KYNA in the plasma and KYNA failed to enter to the brain tissue [11]. The second approach is to inhibit the efflux of KYNA from the brain by using probenecid [12,13] or inhibiting the conversion of KYN to quinolinic acid by using FCE 28833A [14]. Finally, analogues of KYNA can be developed to cross the BBB and to preserve the neuroprotective effect of KYNA in the brain. Beside structural modifications, penetration of KYNA through the BBB can be enhanced by encapsulating it into nanoparticles. Bovine serum albumin (BSA)/KYNA-core was encapsulated by polyallylamine hydrochloride (PAH) as a shell and it had significantly higher permeability than free KYNA [15]. Recent studies have shown that different KYNA derivatives may have as good neuroprotective properties as KYNA itself with possibly better pharmacokinetics. KYNA and its pharmacologically modified analogues proved to have antinociceptive effects at both first and second order sensory neurons in headache models [16]. In an animal model of migraine, administration of KYNA and *N*-(2-*N*,*N*-dimethylaminoethyl)-4-oxo-1H-quinoline-2-carboxamide (SZR-72; also called KYNA-a, KYNAA1 or KYNAA2), decreased electrical trigeminal ganglion (TRG) stimulation-induced PACAP mRNA expression in nucleus trigeminalis caudalis (NTC) [17]. In the inflammation model of TRG activation, overexpression of mitogen-activated protein kinase (MAPK) and NFκB was observed, which could be reduced by intraperitoneally administered KYNA and SZR-72 [18]; furthermore, systematic injection of KYNA and SZR-72 also abolished the elevated levels of c-fos and glutamate in NTC neurons [19]. Greco and colleagues demonstrated the antihyperalgesic effect of KYNA and SZR-72 by attenuating the nitroglycerin-induced neuronal activation in NTC [20]. Beside migraine, excitotoxicity is also known to be involved in the pathology of HD. In a transgenic mouse model of HD, administration of SZR-72 completely prevented the atrophy of the striatal neurons, prolonged the lifetime of mice and ameliorated their hypolocomotion [21]. In order to find new KYNA analogues with better CNS penetration, C-3 aminoalkylated KYNA derivatives bearing amide side-chains have been recently synthesized [22]. In the present study, we present a systematic study on the BBB permeability of the compounds.

## 2. Materials and Methods

### 2.1. General Equipment Used during Syntheses

The ^1^H and ^13^C-NMR spectra were recorded in DMSO-d_6_, solution in 5 mm tubes at room temperature (RT), on a Bruker DRX-500 spectrometer (Bruker Biospin AG, Fällanden, Switzerland) at 500 (1H) and 125 (13C) MHz, with the deuterium signal of the solvent as the lock and TMS as internal standard (1H, 13C). Melting points were determined on a Hinotek X-4 melting point apparatus. Elemental analyses were performed with a Perkin-Elmer 2400 CHNS elemental analyzer (Perkin-Elmer, Waltham, MA, USA). Merck Kieselgel 60F_254_ plates were used for TLC (Merck Millipore, Burlington, MA, USA). Microwave irradiation was carried out using a CEM Discover SP instrument (CEM Corporation, Matthews, NC, USA).

### 2.2. Synthesis of SZR-Compounds

**Figure 2:**
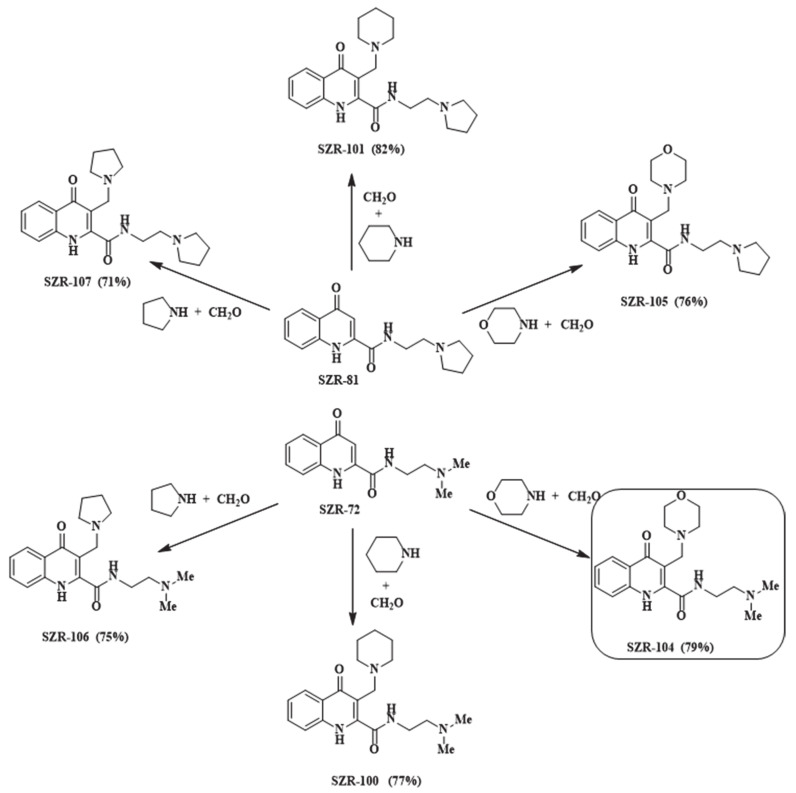
Synthesis of aminoalkylated KYNA amide derivatives.

SZR-100, -101, -104, -105, -106 and -107 were synthesized from KYNA amides 4-oxo-*N*-(2-(pyrrolidin-1-yl)ethyl)-1,4-dihydroquinoline-2-carboxamide (SZR-81) and SZR-72. The reactions were carried out according to the literature method [22], using pyrrolidine, piperidine and morpholine as secondary amines and formaldehyde as aminoalkylating agents (Figure 2).

### 2.3. Synthesis of 8-Hydroxy-3-(Morpholinomethyl)-4-Oxo-1,4-Dihydroquinoline-2-Carboxylic Acid (39B)

**Figure 3:**
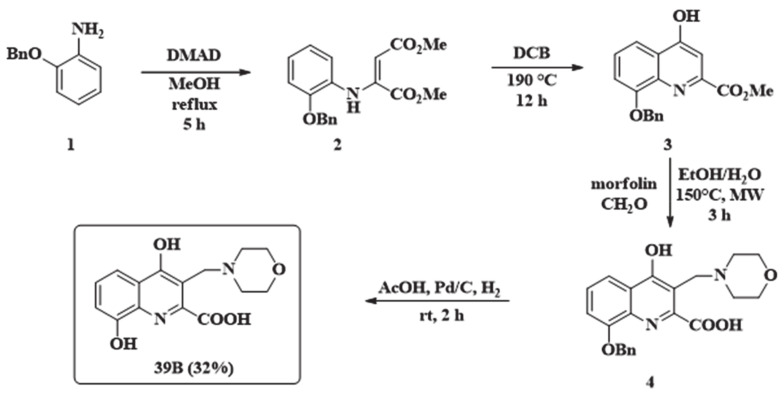
Synthesis of morpholinomethylated xanthurenic acid 39B**.**

39B has already been described in the literature, synthesized by employing benzyl protection of the 8-hydroxy function [23]. During carrying out the described reaction several optimization steps had to be implemented: (i) the enamine formation required longer reaction time; (ii) diphenyl ether as solvent was changed to 1,2-dichlorobenzene for easier work-up; (iii) the aminoalkylation reaction required much higher reaction temperature; (iv) during the reduction step with platinum/carbon catalyst, if the reaction was kept under H_2_ gas as long as described, a decrease in yield was observed. This might be due to the adsorption of the product on the carbon surface that could be eluded with shorter reaction time and a prompt work-up (Figure 3).

2-Benzyloxyaniline (**1**, 213 mg, 1.0 mmol) was placed in a 100 mL reaction kettle with 40 mL MeOH and 1.2 mmol (170 mg) dimethylacetylenedicarboxylate was added to the solution. The reaction was stirred at reflux temperature for 5 h. After evaporation of the solvent, the enamine was purified with column chromatography using *n*-hexane:EtOAc 3:1 as eluent. The ring-closure was carried out in 1,2-dichlorobenzene at 190 °C. After 12 h the reaction was let to cool to room temperature, when the formed precipitate was filtered and washed with 2 × 15 mL EtOAc. The precipitate (**3**) was then put in a pressure resistant 5 mL vial with 74 mg (0.85 mmol) morpholine and 74 mg (0.85 mmol) 35% aqueous formaldehyde. The mixture was irradiated in a microwave reactor for 3 h at 150 °C. After the evaporation of the solvent the residue was crystalized from 10 mL EtOAc. Compound **4** was then dissolved in AcOH (5 mL). In parallel, Pd/C (5%, 50 mg) was suspended in AcOH (10 mL). The mixture was hydrogenated at atmospheric pressure for 2 h. The catalyst was then filtered off and washed with 2 × 10 mL DCM and 2 × 10 mL EtOH. The filtrates were collected and the solvents were removed. The residue was crystalized from 10 mL EtOAc. Overall yield: 97 mg (32%); M.p. 290–295 °C (decomposition). 1H NMR (DMSO-d6); 3.03–3.20 (2H, m); 3.26–3.36 (2H, m); 3.55-3.75 (2H, m); 3.84–4.06 (2H, m); 4.51 (2H, s); 7.12 (1H, d, J = 7.8 Hz); 7.19 (1H, t, J = 7.8 Hz); 7.54 (1H, d, J = 8.1 Hz); 10.27 (1H, brs); 10.95 (1H, brs); 12.13 (1H, brs); 13C NMR (DMSO-d6); 50.3; 50.6; 64.1; 108.9; 115.6; 115.7; 124.6; 125.5; 128.9; 145.0; 147.0; 163.9; 178.0 (Figures S1 and S2).

### 2.4. Isolation of Rat Brain Cells

Primary rat brain endothelial cells (RBECs) were isolated from 2-week old rats. Animals were sacrificed by cervical dislocation. Briefly, after removal of meninges, cerebral cortices were cut into small pieces and digested in two steps with 1 mg/mL collagenase type 2 and 1 mg/mL collagenase/dispase, followed by centrifugation on a percoll gradient (all from Sigma-Aldrich, St. Louis, MO, USA). Isolated microvessels were plated on fibronectin/collagen-coated dishes. Endothelial cells growing out of the microvessels were cultured in DMEM/F12 (Thermo Fisher Scientific, Waltham, MA, USA), 10% plasma-derived serum (PDS, First Link UK Ltd, Birmingham, UK) and growth factors. In the first two days, 4 µg/mL puromycin (Sigma-Aldrich) was added to remove contaminating cells. Cells were frozen in liquid nitrogen and passage 1 cells were further used. Rat brain pericytes were obtained from cerebral microvessels plated onto rat-tail collagen-coated dishes in PM medium (ScienCell, Carlsbad, CA, USA) complemented with 5% fetal bovine serum (FBS) and penicillin/streptomycin. Cells were split into new dishes, frozen in liquid nitrogen and passage 4 cells were further used. Glial cultures (>90% astrocytes) were prepared from newborn rats and cultured on poly-l-lysine-coated surfaces in DMEM (Thermo Fisher Scientific) and 10% FBS (Thermo Fisher Scientific). After reaching confluence, conditioned media was collected twice a week, centrifuged, sterile filtered and kept at −20 °C.

### 2.5. Construction of the In Vitro Blood–Brain Barrier (BBB) Model

Pericytes were plated onto the backside of 12-well semipermeable filters (Corning-Costar Transwell Clear; pore size: 0.4 μm; 1.5 × 10^4^ cells/filter). The next day, RBECs were plated onto the upper surface of the filters. After reaching confluence, the endothelial monolayer was supplied with 550 nM hydrocortisone, 250 μM CPT-cAMP (Sigma-Aldrich) and 17.5 μM RO-201724 (Roche, Basel, Switzerland), and placed into the CellZscope instrument (nanoAnalytics, Münster, Germany) containing glial-conditioned media. TEER was followed until reaching plateau of 150–250 Ohm × cm^2^.

### 2.6. Permeability Assay

Transwell filters containing endothelial cells and pericytes were removed from the CellZscope instrument. Filters were washed with Ringer-HEPES solution (pH = 7.4). Test substances (KYNA, xanthurenic acid, SZR-100, SZR-101, SZR-104, SZR-105, SZR-106, SZR-107 and 39B) were dissolved in Ringer-HEPES and applied in the top compartment in a final concentration of 10 µM. The bottom compartment was loaded with Ringer-HEPES. Filters were placed to 37 °C and gentle shaking (110 rpm) was applied. Samples were taken from the basolateral side after 30 and 60 min. The concentrations of the samples were quantitated as described below and apparent permeability was calculated using the formula: P_app_ = dQ/(dT × A × C_0_), where dQ is the transported amount, dT is the incubation time, A is the surface of filter (1.12 cm^2^), and C_0_ is the initial concentration. Statistical analysis was performed with ANOVA and Bonferroni’s post-hoc test.

### 2.7. Concentration Measurement of KYNA Analogues

The concentrations of the samples were quantitated with an Agilent 1100 HPLC system (Agilent Technologies, Santa Clara, CA, USA), consisting of a binary pump, a micro vacuum degasser, a thermostated column compartment, an automatic liquid sampler, a MS detector (LCMSD VL), and ChemStation data managing software. Chromatographic separations with the HPLC-MS system were performed on a Kinetex C18 column, 100 × 4.6 mm 2.6 μm particle size (Phenomenex, Torrance, CA, USA) with a mobile phase composition of 0.05% aqueous HCOOH/ACN = 90/10 (*v*/*v*), applying isocratic elution. The flow rate and the injection volume were 1 mL/min and 20 µL, respectively. The mass spectrometer was used in positive electrospray ionization mode. The drying gas temperature, drying gas flow rate, nebulizer pressure and capillary voltage were 350 °C, 13 L/min, 60 psi and 3500 V, respectively.

### 2.8. Sample Processing for Determination of KYNA, SZR-104, Xanthurenic Acid and 39B Concentrations

For all analytical steps, LC-MS grade solvents were used. A method for preparation of sample extracts prior to the analysis was the following: 50 µL sample was spiked with 5 µL internal standard (IS) solution containing *N*-(3-(dimethylamino)propyl)-4-hydroxyquinoline-2-carboxamide hydrochloride (SZR-73; 200 nM) in H_2_O/methanol(MeOH)/ammonia (NH_3_) (90/10/0.1 *v*/*v*/*v*) and 5 µL H_2_O/MeOH/NH_3_ (90/10/0.1 *v*/*v*/*v*) solution, then 250 µL ice cold acetone was added in 0.65 mL microcentrifuge tube (Corning-Costar #3206). The sample was vortex mixed for 15 s, centrifuged at 15,000 rpm for 10.5 min at 4 °C degree (Hettich 320R). The 290 µL of the upper layer was transferred to 0.65 mL microcentrifuge tube and evaporated to dryness under nitrogen at ambient temperature (MD 200, Allsheng Instruments, Hangzhou, China). For analysis, the dried extracts were dissolved in 35 μL of H_2_O/MeOH/NH_3_ (90/10/0.1 *v*/*v*/*v*%) and transferred to a 250 μL conical insert. In case of calibration samples, 50 μL Ringer-HEPES buffer sample was spiked with 5 μL given calibration mix containing SZR-104, xanthurenic acid, KYNA and 39B in H_2_O/MeOH/NH_3_ (90/10/0.1 *v*/*v*/*v*) solution, then 5 μL IS and, then 250 μL ice cold acetone was added in 0.65 mL microcentrifuge tube. The following steps are same as described above. The calibration points were the following: 0, 1, 10, 50, 100 and 200 nM for each compound.

### 2.9. Ultrahigh Performance Liquid Chromatography Coupled to Tandem Mass Spectrometry (UHPLC-MS/MS) Parameters for Determination of KYNA, SZR-104, Xanthurenic Acid and 39B Concentrations

UHPLC-MS/MS analysis was performed with using an UHPLC (Nexera, Shimadzu, Kyoto, Japan) coupled to triple quadrupole mass spectrometer (TSQ Fortis, Thermo Fisher Scientific) with an electrospray ionization source (Ion Max, Thermo Fisher Scientific). The UHPLC was controlled using the LabSolutions software version 5.97 SP1 (Shimadzu). Data were acquired and evaluated with Xcalibur 4.2.28.14 software version 4.2.28.14 (Thermo Fisher Scientific). The developed UHPLC-MS/MS method was as follows: ACQUITY UPLC HSS T3 C18 column (100 × 2.1 mm, 1.8 μm, 100 Å, Waters), injection volume 5 μL, and column temperature 50 °C. The isocratic mobile phase composition was H_2_O/MeOH/formic acid (FA) (88/12/0.1 *v*/*v*/*v*). The injector needle was washed with 2-propanol/MeOH/H_2_O/FA (70/25/5/0.1, *v*/*v*/*v*/*v*%) solution after each injection. Mass spectrometer was operating in positive mode using a heated electrospray ionization source with the following conditions: capillary temperature 300 °C, vaporizer temperature 350 °C, spray voltage 3.9 kV, sheath gas flow 10, sweep gas flow 2 and auxiliary gas flow 5 arbitrary unit. The data acquisition was performed in the selected reaction monitoring mode with both Q1 and Q3 resolution FWHM at 2.0. The flow injection optimization was performed in order to determine the proper quantifier and qualifier ions of given protonated precursor ions and optimize the collision energy and tube lens for each analyte (Table 1). A six-point curve of the external calibration was used for the quantification of the compounds, which was based on quantifier ion of analyte/quantifier ion of SZR-73 peak area ratios vs concentration [24].

## 3. Results and Discussion

As KYNA has poor CNS penetration, novel strategies are needed to take advantage of its important neuroprotective effects. Among the several possible approaches [9], two strategies seem to be the most suitable in this respect. First, the use of vector-mediated drug delivery through the brain endothelium, especially nanocarriers, which are admittedly among the most promising tools to overcome the BBB [9]. Our research consortium has been successfully working in this field as well [15]. Second, the synthetic approach, i.e., chemical modification of KYNA to obtain compounds with similar biological effects but significantly improved ability to cross the BBB-forming cerebral endothelial cells.

Here, we applied this second strategy and we designed new compounds (aminoalkylated KYNA amide derivatives). The compounds investigated and thus the modifications carried out on the KYNA skeleton were chosen based on a previous study on C-3 substituted KYNA derivatives [23]. Through the use of different secondary amines (piperidine, pyrrolidine) and also morpholine, we aimed to investigate the BBB penetration altering effect of the aminoalkyl function formed during the modified Mannich reactions.

**Figure 4:**
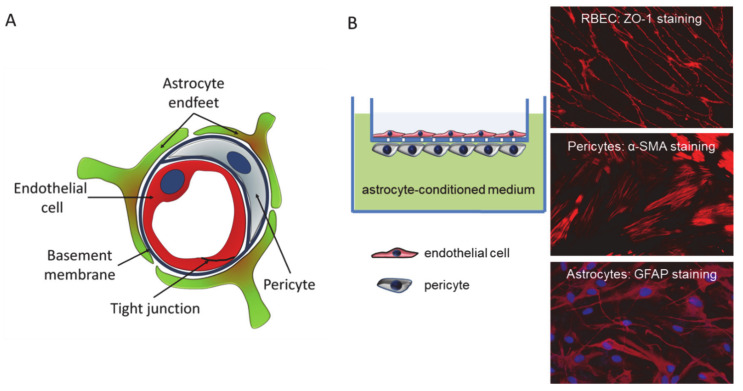
The in vitro BBB model. (**A**) Schematic representation of the cross-section of a brain capillary, showing the cellular composition. (**B**) The in vitro BBB model consists of cerebral endothelial cells cultured on filter inserts having pericytes on the bottom surface. Filters containing endothelial cells and pericytes are emerged in a well containing astrocyte-conditioned media. Right panels show representative immunofluorescence staining of respective cellular markers.

We first assessed the permeability of the newly synthesized compounds using an in vitro model system, which mimics the in vivo anatomical structure of the BBB (Figure 4) and is suitable for drug testing [25,26].

**Figure 5:**
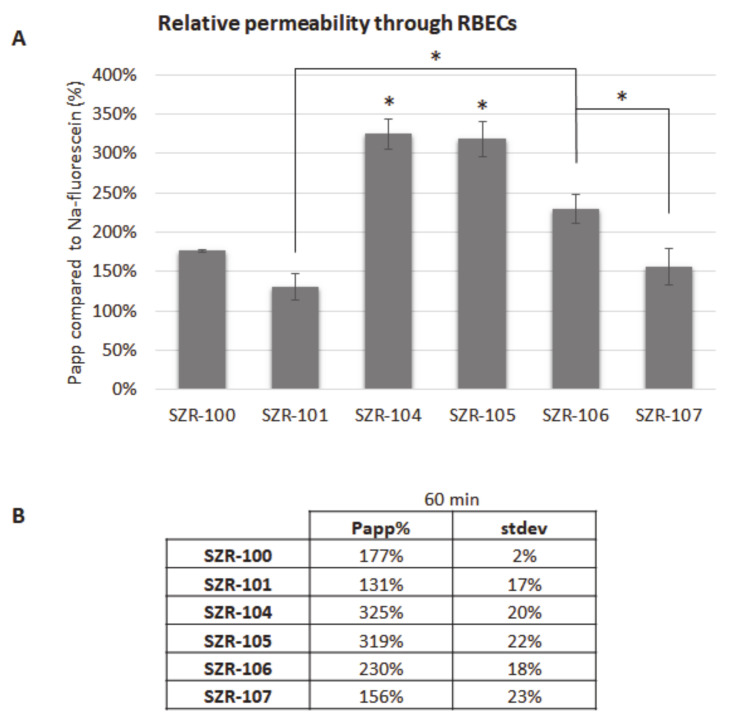
Penetration of KYNA analogues through the BBB. (**A**) Permeability of KYNA derivates after 60 min compared to sodium-fluorescein. N = 2, average ± SD (ANOVA and Bonferroni’s post hoc test). * *p* < 0.05 (SZR-104 and SZR-105: significant difference compared to all other groups; significant difference between SZR-106 and SZR-101 or SZR-107). (**B**) Permeability coefficients of KYNA analogues after 60 min compared to sodium-fluorescein.

KYNA analogues (SZR-100, SZR-101, SZR-104, SZR-105, SZR-106 and SZR-107) were applied in the top/blood compartment in a final concentration of 10 µM. Samples were collected from the bottom/brain compartment after 60 min. Sodium-fluorescein was used as a control compound, since it is a small molecular tracer for the indication of cellular junctions’ integrity and restricted paracellular transport between cerebral endothelial cells. All of the above-mentioned analogues crossed the BBB more efficiently than sodium-fluorescein. Furthermore, the permeability of SZR-104 and SZR-105 was significantly higher than the permeability of other analogues (Figure 5).

**Figure 6:**
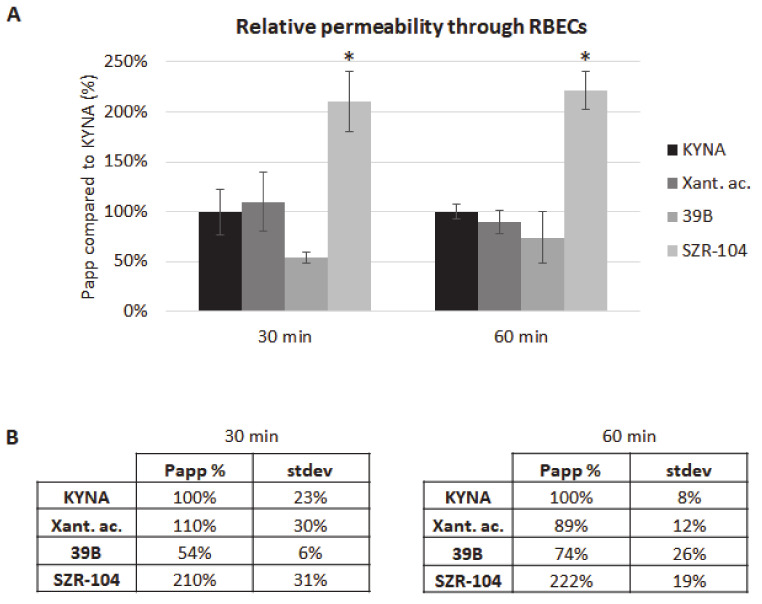
Penetration of SZR-104 through the BBB**. (A)** Permeability of SZR-104 after 30 and 60 min compared to KYNA, xanthurenic acid and its analogue, 39B. N = 3, average ± SD. * *p* < 0.01 (SZR-104: significant difference compared to all other groups, in both time-points). **(B)** Permeability coefficients of SZR-104, xanthurenic acid and 39B after 30 and 60 min compared to KYNA.

SZR-104 not only had the highest permeability in our preliminary study, but previous results suggested that it had important biological effects. In a recent study, human lymphoma U-937 cells were infected with Staphylococcus aureus to induce cytokine production and then were treated with KYNA and its newly synthesized analogues (SZR-104, SZR-105 and SZR-109). The analogues reduced tumor necrosis factor-α (TNF-α) production and increased the mRNA expression of the anti-inflammatory Tumor necrosis factor-Stimulated Gene-6 (*TSG-6*) [27]. In addition, an in vivo electrophysiological study has demonstrated that systemic administration of SZR-104 decreased population spike activity in the hippocampus, and additionally provided protection against pentylenetetrazol-induced epileptiform seizures [28]. Therefore, we continued our study and focused on the characterization of SZR-104. We compared its permeability with that of KYNA, xanthurenic acid and its analogue, 39B, which is under patent protection [23]. SZR-104 had a significantly higher permeability through the in vitro BBB model than KYNA, xanthurenic acid or 39B both at 30 min and at 60 min time points. Differences among permeability of KYNA, xanthurenic acid and 39B were not statistically significant (Figure 6). Our results indicate that SZR-104 has a much higher permeability through the BBB than free KYNA.

## 4. Conclusions

Based on our systematic investigation, SZR-104 and SZR-105 both showed high BBB penetration (with the former having the highest apparent permeability) compared to the other derivatives tested. In line with this comparison, we hypothesize that aminoalkylation in C-3 facilitates BBB penetration with the morpholinomethyl functional group showing the best results. These results are in line with in vivo data showing that peripherally administered SZR-104 can reach sufficient concentration in the brain, since it is able to inhibit epileptiform activity [28]. In conclusion, SZR-104 is a promising neuroprotective candidate drug.

## Figures and Tables

**Table 1:** UHPLC-MS/MS parameters for selected reaction monitoring transitions in positive mode.Xant. ac = xanthurenic acid.

Compound	Retention Time (min)	Retention Time Window (min)	Precursor Ion (*m/z*)	Type of Production	Product Ion (*m/z*)	Collision Energy (eV)	RF Lens (V)
SZR-104	1.2	2.0	359.2	quantifier	130.1	54	78
SZR-104	1.2	2.0	359.2	qualifier	272.0	15	78
Xant. ac.	3.5	1.0	206.0	quantifier	131.9	30	113
Xant. ac.	3.5	1.0	206.0	qualifier	160.0	20	113
KYNA	4.0	1.0	190.1	quantifier	144.0	19	55
KYNA	4.0	1.0	190.1	qualifier	172.0	13	55
39B	6.0	1.0	305.1	quantifier	174.0	22	80
39B	6.0	1.0	305.1	qualifier	261.0	10	80
SZR-73	6.5	1.0	274.2	quantifier	144.0	37	65
SZR-73	6.5	1.0	274.2	qualifier	229.0	17	65

## Data Availability

The data presented in this study are available on request from the corresponding author.

## Supplementary Materials

The following are available online at https://www.mdpi.com/1999-4923/13/1/61/s1, Figures S1 and S2: NMR spectra of synthesized compounds.
