# Supplementary material for: SZR-104, a Novel Kynurenic Acid Analogue with High Permeability through the Blood–Brain Barrier
Source: Pharmaceutics. 2021 Jan 5;13(1):61. doi: 10.3390/pharmaceutics13010061 (PMC7824826; doi:10.3390/pharmaceutics13010061)
Supplement: Supplementary file 1 [file pharmaceutics-13-00061-s001.pdf]

Supplementary Material:

SZR-104, a Novel Kynurenic Acid Analogue with High Permeability through the Blood–Brain Barrier

Contents: 1. ^{1}H NMR and ^{13}C NMR spectra of 39B

8-Hydroxy-3-(morpholinomethyl)-4-oxo-1,4-dihydroquinoline-2-carboxylic acid (39B)

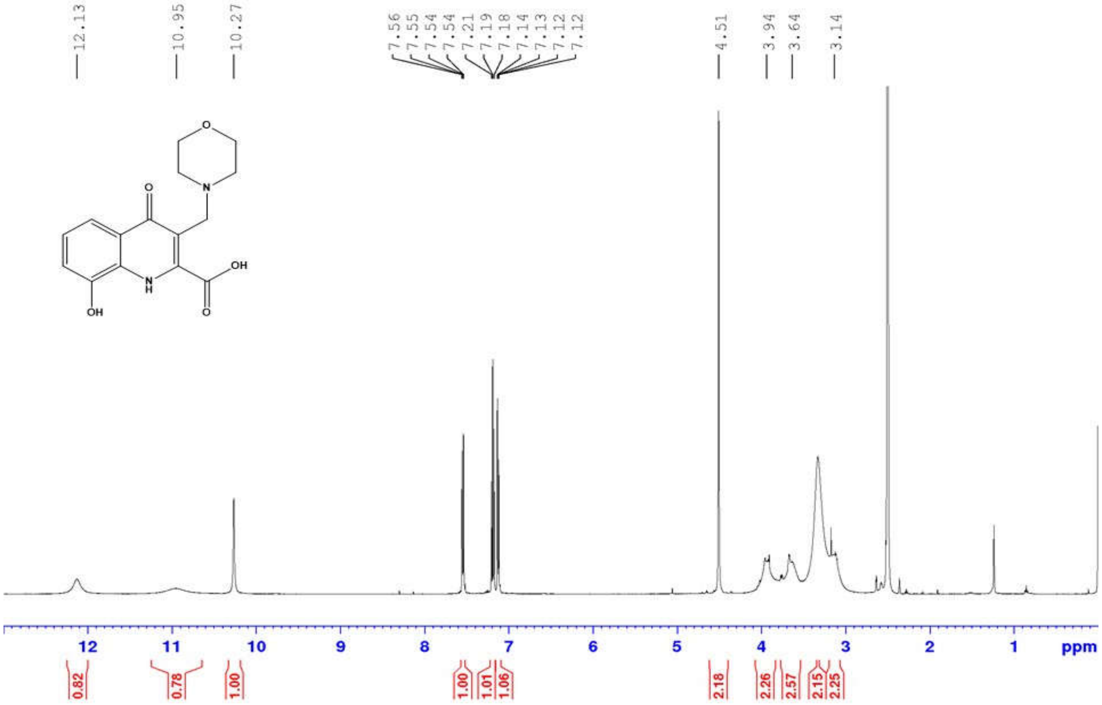

Figure 1. ^{1}H-NMR spectrum of 39B.

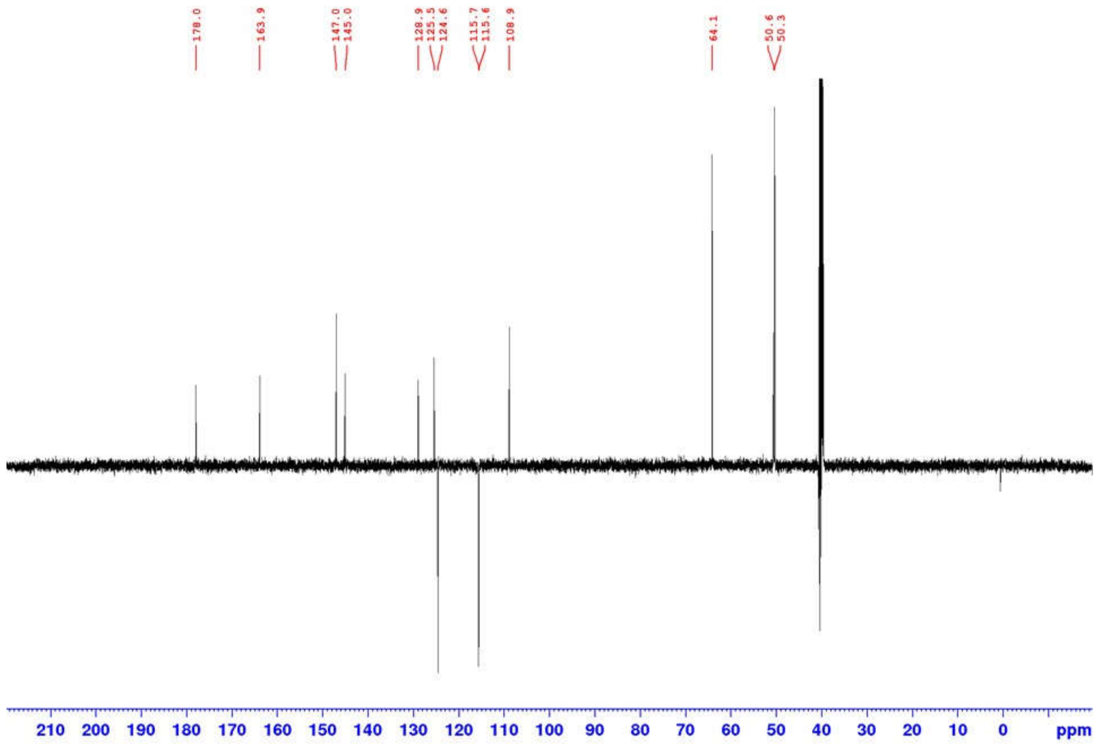

Figure S2. ^{13}C-NMR spectrum of 39B
